# Non-Motor Symptom Management: Insights into Adherence to Treatment Guidelines in Parkinson’s Disease Patients

**DOI:** 10.3233/JPD-230263

**Published:** 2024-03-05

**Authors:** Carin Janz, Jonathan Timpka, Kristina Rosqvist, Gesine Paul, Alexander Storch, Per Odin

**Affiliations:** a Department of Clinical Sciences Lund, Division of Neurology, Lund University, Lund, Sweden; b Department of Neurology, Rehabilitation Medicine, Memory and Geriatrics, Skåne University Hospital, Lund, Sweden; c Department of Neurology, University of Rostock, Rostock, Germany; d German Center for Neurodegenerative Diseases (DZNE) Rostock-Greifswald, Rostock, Germany

**Keywords:** Movement disorder, Parkinson’s disease, disease management, guidelines, symptoms

## Abstract

**Background:**

Non-motor symptoms (NMS) reduce quality of life in Parkinson’s disease (PD) patients, who experience three times more NMS than individuals without PD. While there are international and national NMS treatment guidelines, their implication in clinical practice remains unclear.

**Objective:**

This study aimed to investigate the adherence to pharmacological NMS treatment guidelines in patients with mild to moderately severe PD.

**Methods:**

220 PD patients with ≥1 NMS based on the Non-Motor Symptom Questionnaire and a Hoehn and Yahr stage ≤4 were randomly selected from the Swedish Parkinson registry and screened for inclusion. NMS were evaluated using the International Parkinson and Movement Disorder Society–Non-Motor Rating Scale (MDS-NMS), Parkinson’s Disease Sleep Scale 2, Epworth Sleepiness Scale, and Hospital Anxiety and Depression Scale. Treatment was compared with Swedish national guidelines and international guidelines from the MDS Evidence-Based Medicine Committee.

**Results:**

Among 165 included patients, the median number of NMS was 14, and in median 7 symptoms were estimated to require treatment. The most common NMS requiring treatment were pain (69%) and urinary problems (56%). Treatment of depression and constipation demonstrated the highest adherence to guidelines (79% and 77%), while dysphagia and excessive daytime sleepiness exhibited the lowest adherence (0% and 4%). On average, only 32% of NMS were treated in accordance with guidelines.

**Conclusions:**

Adherence to pharmacological guidelines for NMS in patients with mild to severe PD was low. This study highlights the need for improved evaluation and treatment of NMS to enhance symptom management and quality of life among PD patients.

## INTRODUCTION

Parkinson’s disease (PD) is the second most common movement disorder [1], and 98% of PD patients experience at least one non-motor symptom (NMS) [2]. On average, patients experience eight of the NMS monitored by the Non-motor symptom questionnaire, which is three times more than age-matched controls [3]. NMS involves for example cognitive dysfunction [1], constipation, excessive daytime sleepiness, sleep disturbances, depression, anxiety, pain, urinary dysfunction, and orthostatic dysfunction [4]. The exact causes of NMS are likely diverse and not fully understood, but a dysfunction of both dopaminergic and non-dopaminergic neurotransmitter systems are thought to play significant roles in their development [4]. Also, NMS can be side effects of ongoing treatment [4]. For example, degeneration of peripheral autonomic neurons, the medulla oblongata and the olfactory bulb contribute to the development of NMS [5].

Many NMS respond to dopaminergic therapy [6], particularly when NMS are associated with motor fluctuations, such as pain or depression during “Off” state [7, 8]. However, some NMS, like orthostatic hypotension and dopamine dysregulation syndrome, can worsen with the use of dopaminergic treatment [6]. The Swedish National Board of Health and Welfare [9, 10], The Swedish Movement Disorder Society (Swemodis) [7], and The International Parkinson and Movement Disorder Society (MDS) Evidence-Based Medicine committee provide evidence-based treatment guidelines for managing NMS [11]. A recent study assessed the adherence to NMS treatment guidelines in late stage PD patients with a Hoehn and Yahr stage ≥4, using MDS guidelines from 2019, Swemodis guidelines from 2019, and the Swedish National Board of Health and Welfare guidelines from 2016 [12]. The study demonstrated a high prevalence and severity of NMS in advanced stages of PD and emphasized the importance of comprehensive screening and effective management of NMS in patients with late-stage PD. However, it is still unknown to what extent the international and national NMS treatment guidelines are followed for patients with mild to severe PD. This information is crucial for gaining insights into the adherence to NMS treatment guidelines across the entire patient group.

NMS significantly impacts the quality of life for individuals with PD [13, 14], and imposes a considerable financial burden on society due to increased institutional care admissions and premature retirement [8]. Therefore, detecting NMS early and providing appropriate treatment is essential for both individuals with PD and society. However, it appears that clinicians often overlook NMS and fail to inquire about the patients’ problems [13]. Investigating adherence to treatment guidelines can help address this issue by raising awareness of potential gaps and areas for improvement in NMS management. Understanding how NMS guidelines are followed is essential for improving both the guidelines themselves and the NMS treatment approaches. By assessing the current implementation of NMS treatment guidelines, we can work towards improving patient care and outcomes.

Consequently, the aim of this study was to investigate the adherence to pharmacological NMS treatment guidelines among PD patients with a disease severity spanning from mild to severe stages, who were able to walk or stand unassisted (i.e., Hoehn and Yahr stage ≤4).

## MATERIALS AND METHODS

### Participation criteria

This descriptive study included patients diagnosed with idiopathic PD according to the MDS criteria [15]. Participants with a Hoehn and Yahr stage of ≤4 [16], and at least one NMS according to the Non-Motor Symptoms Questionnaire (NMSQ, ≥1 positive answer) were eligible for inclusion [17]. Additionally, individuals were required to be capable of providing informed consent and completing questionnaires. Patients exhibiting clinical signs of secondary or atypical parkinsonism, inability to complete patient questionnaires due to severe dementia or other conditions affecting their ability to consent or adhere to the study protocol were excluded from participation.

### Participant selection

A total of 220 patients were randomly selected from the Swedish Parkinson registry (ParkReg). ParkReg is a national Parkinson patient registry belonging to the Swedish Neuroregistries, and coverage in the Scania region is around 70%. Patients were required to be affiliated with a Neurology department in Scania, Sweden; have scored at least one point on the NMSQ in ParkReg within the previous two years and have a Hoehn and Yahr score of ≤4. The selection aimed for an equal distribution between males and females.

### Instruments and assessments

The international Parkinson and Movement Disorder Society–Non-Motor Rating Scale (MDS-NMS) was utilized to assess the NMS experienced by the patients [18]. The questionnaire, completed by the rater, assesses the frequency and severity of 13 domains of NMS, comprising a total of 52 items. The scale was administered via telephone, with the questions translated into Swedish beforehand by the investigator, CJ. In addition, the patients received three questionnaires by mail to complete and return. The scales they answered at home were the Epworth Sleepiness Scale (ESS) to assess daytime sleepiness [19], the Parkinson’s Disease Sleep Scale 2 (PDSS-2) to assess sleep [20], and the Hospital Anxiety and Depression Scale (HADS) to assess anxiety and depression [21].

### Study design

#### Prescreening

Before contacting the 220 patients selected from the registry, a pre-screening was conducted based on the ParkReg and their medical records to ensure that subjects were neither deceased nor should be excluded for any other reason.

#### Patient contact

The study information and questionnaires (ESS, PDSS-2, and HADS) were sent to all eligible patients who were not excluded during the pre-screening process. The patients signed the informed consent, completed the questionnaires, and provided information about the onset of their motor symptoms, the onset of their first NMS, and the year of their diagnosis. Thereafter, they returned the completed materials. Furthermore, they were contacted by telephone to participate in the rater-administered MDS-NMS scale.

#### Treatment information

Information regarding treatment was collected from patients’ medical records, with any uncertainties clarified through direct patient inquiries. In cases of any ambiguity or uncertainty, the patients were also directly asked about their treatment.

#### Cut-off values

To determine if a patient was symptomatic and hence required treatment for a specific symptom, the following criteria were used: if there was a specific treatment available for an item within a domain, the patient needed to score ≥6 points on that specific item in the MDS-NMS domain. If there was no specific treatment for the item but for the entire domain, the patient needed to score ≥6 points on any of the items within that domain. Additionally, specific criteria were established for insomnia, daytime sleepiness, depression, and anxiety.

For insomnia, the patient needed to score ≥6 points on the insomnia item (domain K question 1) in the MDS-NMS and ≥15 points on the PDSS-2 [22]. Regarding daytime sleepiness, a score of ≥6 points on the daytime sleepiness item (domain K question 3) in the MDS-NMS and ≥13 points on the ESS were required [23]. To be deemed in need of treatment for depression, the patient had to score ≥6 points on any item within the depression domain (A) and ≥8 points on the HADS for depression. Similarly, for anxiety, a score of ≥6 points on any item within the anxiety domain (B) and ≥8 points on the HADS for anxiety were necessary [24].

The patients were defined to have a symptom if they had ≥1 point on that MDS-NMS item if there was a specific treatment for the item. If there was no designated treatment for the specific item but for the entire domain, it was defined as ≥1 points on any item within that domain. The patient had the symptom insomnia if they had ≥1 point on the insomnia item (domain K question 1) in the MDS-NMS and ≥1 point on the PDSS-2. They were defined to have symptoms of daytime sleepiness if they had ≥1 point on the daytime sleepiness item (domain K question 3) in the MDS-NMS and ≥1 point on the ESS. They had the symptom depression if they had ≥1 points on any item within domain A in MDS-NMS and ≥1 points on the HADS for depression. Similarly, for the symptom anxiety,≥1 point on any item within domain B and ≥1 on the HADS for anxiety was required.

#### Treatment guidelines

The study assessed adherence to both national and international NMS treatment guidelines. The national guidelines encompassed the Swemodis guidelines from 2022 and the treatment guidelines from the Swedish National Board of Health and Welfare from 2022 [7, 9, 10]. The international guidelines included the MDS guidelines from 2019 [11]. NMS that are a part of the MDS-NMS scale and for which treatment guidelines exist according to any of these guidelines were included in the study. The Swedish National Board of Health and Welfare guidelines utilize the term “should be used” for priorities 1–4, “can be used” for priorities 5–7, and “can be used in exceptional cases” for priorities 8–10 [7, 10]. For this study, treatments falling within priorities 1–7 or those without a specific priority were considered appropriate treatments. The Swemodis guidelines do not have a grading. The MDS categorize treatments into “clinically useful”, “possibly useful”, “unlikely useful”, “not useful”, or “investigational” [11]. In this study, treatments falling into the categories of clinically useful or possibly useful were included.

First, we assessed the NMS experienced by the patients, identified those requiring treatment, and examined the respective treatments. Subsequently, we compared actual treatments with the recommended guidelines. To do this, we listed all NMS assessed in the MDS-NMS and recommended treatments in a table. For each patient, we checked whether they received correct treatment for the NMS that they experienced as per the guidelines. While the specific indications for each treatment were unknown, we considered the guidelines to be followed if the patient was on medication recommended for a NMS that the patients were symptomatic for.

### Statistical analysis

Descriptive statistics were used to investigate how patients were treated and the adherence to treatment guidelines. Data is presented as means±standard deviations (SD), median and interquartile range (IQR, q1-q3), or frequencies and percentages. Levodopa equivalent doses (LED) were calculated following the method described by Tomlinson et al. [25]. Descriptive analyses were conducted using Microsoft Excel.

### Ethics review

The study was approved by the Swedish Ethical Review Authority (Dnr 022-05274-01) and performed in line with the principles of the Declaration of Helsinki. Written informed consent was obtained from the patients before participating in the study.

## RESULTS

### Participant selection

220 patients were prescreened for the study. Out of these, eight were found to be deceased, and one no longer resided in Sweden. Among the remaining 211 patients who were contacted, it was discovered that 12 resided in an elderly care facility and were deemed unfit to participate according to a relative or a caretaker. Additionally, 34 patients declined to participate in the study. Consequently, a total of 165 participants were included in the study.

### Clinical and demographic information

On average, participants were 71±9 years old, it had been 11±7 years since their diagnosis, and the LED was 890±490 mg/day (Table 1). The median Hoehn and Yahr stage was 2 (IQR: 2-3) with 12% of participants classified as stage one, 37% as stage two, 35% as stage three, and 13% as stage four.

**Table 1:** Clinical and demographic characteristics of study cohort^a^

Gender (*n*, %)
Male	80 (48%)
Female	85 (52%)
Participants from different neurological departments (*n*)	165
Lund (*n*, %)	132 (80%)
Ystad (*n*, %)	2 (1%)
Helsingborg (*n*, %)	3 (2%)
Ängelholm (*n*, %)	14 (8%)
Kristianstad (*n*, %)	14 (8%)
Age, y (mean±SD)	71±9
Age at first motor symptom, y (mean±SD)	58±13
Age at first non-motor symptom, y (mean±SD)	59±13
Age at diagnosis, y (mean±SD)	60±12
PD duration, y since diagnosis (mean±SD)	11±7
LED^b^ (mean±SD)	890±490
NMSQ^c^ total score (median, IQR)	10 (6–14)
Hoehn and Yahr stage (median, IQR)	2 (2–3)
Stage 1 (*n*, %)	24 (15%)
Stage 2 (*n*, %)	61 (37%)
Stage 3 (*n*, %)	58 (35%)
Stage 4 (*n*, %)	22 (13%)
ESS^d^ total score (median, IQR)	8 (5–13)
≥13 (*n*, %)	47 (28%)
PDSS 2^e^ total score (median, IQR)	17 (11–26)
≥15 (*n*, %)	92 (56%)
HADS^f^ total score (median, IQR)	10 (5–15)
Anxiety total score	6 (3–9)
≥8 (*n*, %)	55 (33%)
Depression total score	4 (2–7)
≥8 (*n*, %)	38 (23%)
MDS-NMS^g^ total score (median, IQR)	98 (71–140)

^a^Values are reported as median and interquartile rage (IQR), mean±standard deviation (SD) or number (n) and percentages. 165 participants are included in total. ^b^LED, Levodopa equivalent dose, mg/day. ^c^NMSQ, Non-motor symptom questionnaire. ^d^Epworth Sleepiness Scale. ESS total score ≥13 indicates moderate daytime sleepiness. ^e^Parkinson’s disease sleep scale 2. Total score ≥15 indicates insomnia. ^f^Hospital Anxiety and Depression Scale. ≥8 points on the HADS for depression indicates depression and ≥8 points on the HADS for anxiety indicates anxiety. ^g^Movement Disorder Society Non-Motor Rating Scale.

### NMS among participants

The median MDS-NMS total score was 98 (IQR: 71–140), with participants experiencing a minimum of five NMS and a median of 14 different NMS (Table 2). Among the participants, all but two (*n* = 163) were symptomatic (as defined under “cut-off values” in the methods section) and hence estimated to require treatment for at least one symptom. In median, participants required treatment for seven different NMS.

**Table 2:** Non-motor symptoms according to the MDS-NMS scale^a^

	MDS-NMS score	Symptom present^b^	≥6 points^c^
	(Median, IQR)	(*n*, %)	(*n*, %)
A. Depression, total	6 (1–16)	127 (77%)	88 (53%)
1. Sad or depressed	4 (0–6)	102 (62%)	55 (33%)
2. Difficulties in experiencing pleasure	0 (0–4)	70 (42%)	40 (24%)
3. Hopelessness	0 (0–4)	63 (38%)	30 (18%)
4. Negative thoughts about yourself	0 (0–4)	62 (38%)	20 (12%)
5. Felt that life is not worth living	0 (0–0)	31 (19%)	18 (11%)
B. Anxiety, total	6 (1–14)	129 (78%)	92 (56%)
1. Worried	4 (0–6)	104 (63%)	52 (32%)
2. Nervous	0 (0–4)	74 (45%)	35 (21%)
3. Panic or anxiety attacks	0 (0–0)	35 (21%)	19 (12%)
4. Social phobia	0 (0–4)	73 (44%)	39 (24%)
C. Apathy, total	4 (0–10)	112 (68%)	72 (44%)
1. Reduced motivation to start day-to day activities	0 (0–4)	81 (49%)	39 (24%)
2. Reduced interest in talking to people	0 (0–4)	59 (36%)	24 (15%)
3. Reduction in experiencing emotions	0 (0–4)	63 (38%)	29 (18%)
D. Psychosis, total	1 (0–4)	83 (50%)	39 (24%)
1. Passage or presence phenomena	0 (0–2)	67 (41%)	18 (11%)
2. Illusions	0 (0–0)	36 (22%)	10 (6%)
3. Hallucinations	0 (0–0)	33 (19%)	22 (13%)
4. Delusions	0 (0–0)	11 (6%)	6 (4%)
E. Impulse Control and Related Disorders, total	0 (0–0)	23 (14%)	6 (4%)
1. Impulse control disorders	0 (0–0)	12 (7%)	3 (2%)
2. Other compulsive behaviors	0 (0–0)	5 (3%)	1 (1%)
3. Punding	0 (0–0)	6 (4%)	1 (1%)
4. Dopamine dysregulation syndrome	0 (0–0)	4 (2%)	0 (0%)
F. Cognition, total	12 (6–19)	154 (93%)	130 (79%)
1. Difficulties remembering things	4 (1–6)	136 (82%)	53 (32%)
2. Difficulties learning new things	0 (0–4)	79 (48%)	30 (18%)
3. Difficulties keeping focus or attention	1 (0–4)	84 (51%)	41 (25%)
4. Difficulties finding words or expressing ideas	4 (0–6)	122 (74%)	54 (33%)
5. Difficulties with executive abilities	0 (0–0)	20 (12%)	10 (6%)
6. Difficulties with visuospatial abilities	0 (0–0)	33 (20%)	10 (6%)
G. Orthostatic Hypotension, total	4 (0–9)	96 (58%)	69 (42%)
1. Lightheaded or fainted when changing position	0 (0–4)	69 (42%)	34 (21%)
2. Dizziness or weakness upon standing	1 (0–6)	87 (53%)	50 (30%)
H. Urinary, total	6 (2–13)	131 (79%)	96 (58%)
1. Urinary urgency	4 (0–6)	123 (75%)	81 (49%)
2. Urinary frequency	0 (0–4)	58 (35%)	27 (16%)
3. Nocturia	0 (0–2)	48 (29%)	27 (16%)
I. Sexual, total	0 (0–0)	49 (30%)	23 (14%)
1. Decreased sex drive or interest	0 (0–2)	47 (28%)	14 (8%)
2. Difficulties with sexual arousal or performance	0 (0–0)	18 (11%)	14 (8%)
J. Gastrointestinal, total	8 (4–13)	149 (90%)	113 (68%)
1. Drooling	2 (0–6)	100 (61%)	48 (29%)
2. Difficulties wallowing	0 (0–4)	65 (39%)	22 (13%)
3. Nausea or sick in stomach	0 (0–0)	40 (24%)	15 (9%)
4. Constipation	2 (0–6)	107 (65%)	66 (40%)
K. Sleep and wakefulness, total	11 (5–19)	154 (93%)	122 (74%)
1. Insomnia	4 (0–6)	107 (65%)	70 (42%)
2. REM^*d*^ sleep behavior	1 (0–4)	95 (57%)	30 (18%)
3. Excessive daytime sleepiness	1 (0–4)	102 (62%)	36 (22%)
4. Restlessness	1 (0–6)	82 (50%)	53 (32%)
5. Periodic limb movements	0 (0–0)	27 (16%)	12 (7%)
6. Snoring or difficulty breathing	0 (0–0)	15 (9%)	5 (3%)
L. Pain, total	10 (4–16)	140 (85%)	119 (72%)
1. Muscle, joint, back pain	6 (4–9)	130 (79%)	108 (65%)
2. Deep or dull pain	0 (0–0)	28 (17%)	20 (12%)
3. Dystonia	2 (0–6)	92 (56%)	49 (30%)
4. Other pain	0 (0–0)	32 (19%)	25 (15%)
M. Other, total	18 (12–27)	157 (92%)	141 (83%)
*1. Weight loss*	0 (0–4)	46 (28%)	33 (20%)
*2. Decreased smell*	8 (0–12)	116 (70%)	97 (59%)
*3. Physical fatigue*	3 (0–6)	102 (62%)	62 (38%)
*4. Mental fatigue*	1 (0–6)	90 (55%)	57 (35%)
*5. Excessive sweating*	0 (0–4)	67 (40%)	39 (24%)
MDS-NMS total score	98 (71–140)

^a^MDS-NMS = The international Parkinson and Movement Disorder Society –Non-Motor rating Scale (0 –832, higher scores indicate worse symptoms). Each letter (A - M) represents a domain, and each part within that domain is an item. Each item is scored based on the frequency (0 –4, higher scores indicate more frequent occurrence) multiplied by the severity (0 –4, higher scores indicate greater severity). The domain score is obtained by summing the severity x frequency scores of each item within the domain. Frequency score ranges from 0 to 4, where 0 represents ‘Never,’ 1 represents ‘Rarely’ (≤10% of the time), 2 represents ‘Sometimes’ (11–25% of the time), 3 represents ‘Frequently’ (26–50% of the time), and 4 represents ‘Majority of the time’ (≥51% of the time). The severity score also ranges from 0 to 4, where 0 is ‘Not present’ (only if frequency score is 0), 1 represents ‘Minimal’ (no distress or disturbance to the patient or caregiver), 2 represents ‘Mild’ (minor distress or disturbance), 3 represents ‘Moderate’ (considerable distress or disturbance), and 4 represents ‘Severe’ (major distress or disturbance). Values are reported as median and first and third quartiles (IQR) or as numbers and percentages. ^b^Number of participants with at least one point on that item/domain on MDS-NMS. ^c^Number of participants with ≥6 point on that item/domain on MDS-NMS. A cutoff score of ≥6 was utilized to identify patients requiring treatment for a particular symptom. If there was no distinct treatment for that specific item but for the entire domain, the patient needed a score of ≥6 points on any of the items within that domain. ^d^Rapid eye movement sleep behavior disorder.

Among the participants who scored ≥6 points on the “insomnia” question (domain K question 1 on MDS-NMS), 55 out of 70 also obtained ≥15 points on the PDSS-2, indicating they were experiencing symptomatic insomnia. Among those with ≥6 points on the “excessive daytime sleepiness” question (domain K question 3 on MDS-NMS), 28 out of 36 scored ≥13 points on the ESS, indicating symptomatic daytime sleepiness. Furthermore, among participants who scored ≥6 points on any of the questions in MDS-NMS domain A (“Depression”), 29 individuals also scored ≥8 points on the HADS concerning depression, meaning they were symptomatic for depression. Additionally, 44 participants scored ≥8 points on the HADS concerning anxiety and ≥6 points on any of the questions in MDS-NMS domain B (“Anxiety”), indicating a need for anxiety treatment. The number of symptomatic participants is displayed in Table 3.

**Table 3:** Adherence to the treatment guidelines for non-motor symptoms based on the Swedish National Board of Health and Welfare’s guidelines, the Swedish Movement Disorder Society’s guidelines and the Parkinson and Movement Disorder Society guidelines^a^

Non-motor symptom	Symptomatic^b^	Adherence to	Adherence to	Adherence to
	(*n*, %)	Swedish guidelines^c^	MDS guidelines^d^	Swedish and/or MDS
		(*n*, %)	(*n*, %)	guidelines^e^ (*n*, %)
Depression	29 (18%)	22 (76%)	22 (76%)	23 (79%)
Anxiety	44 (27%)	25 (57%)	––	25 (57%)
Apathy	58 (35%)	4 (7%)	4 (7%)	4 (7%)
Psychosis	32 (19%)	5 (16%)	5 (16%)	5 (16%)
Cognitive impairment	91 (55%)	6 (7%)	6 (7%)	6 (7%)
Orthostatic hypotension	55 (33%)	5 (9%)	4 (7%)	5 (9%)
Urinary problems	93 (56%)	31 (33%)	2 (2%)	31 (33%)
Sexual dysfunction^f^	Male: 16 (20%)	––	6 (38%)	6 (38%)
	Female: 6 (7%)
Drooling of saliva	48 (29%)	16 (33%)	3 (6%)	16 (33%)
Dysphagia	22 (13%)	0 (0%)	––	0 (0%)
Nausea or stomach sick	15 (9%)	8 (53%)	1 (7%)	8 (53%)
Constipation	66 (40%)	51 (77%)	43 (65%)	51 (77%)
Insomnia	55 (33%)	33 (60%)	15 (27%)	34 (62%)
REM^g^ sleep disturbances	30 (18%)	9 (30%)	––	9 (30%)
Excessive daytime sleepiness	28 (17%)	––	1 (4%)	1 (4%)
Restless leg syndrome	53 (32%)	16 (30%)	––	18 (33%)
Pain	114 (69%)	43 (38%)	4 (4%)	43 (38%)
Dystonia	49 (30%)	6 (12%)	––	6 (12%)
Fatigue^h^	All fatigue: 76 (46%)	––	15 (20%)	15 (20%)
	Mentally: 57 (35%)
	Physically: 62 (38%)
Excessive sweating	39 (24%)	10 (26%)	––	10 (26%)

^a^The Swedish guidelines incorporate both the Swedish National Board of Health and Welfare guidelines and the Swedish Movement Disorder Society’s treatment guidelines. The absence of guidelines for a symptom is denoted by “––”. *n* = number. ^b^Number of patients who were considered to require treatment for the symptom, % of total cohort (*n* = 165). Patients with ≥6 points on that item on the International Parkinson and Movement Disorder Society - Non-Motor Rating Scale (MDS-NMS) were considered to require treatment for that symptom. If there was no designated treatment for a specific item but for the entire domain, the patient needed to score ≥6 points on any item within that domain. To be considered treatment for insomnia, a patient needed ≥6 points on the insomnia item (domain K question 1) in the MDS-NMS and ≥15 points on the PDSS-2. Daytime sleepiness required ≥6 points on the daytime sleepiness item (domain K question 3) in the MDS-NMS and ≥13 points on the ESS. In the depression category, ≥6 points on any item within domain A in MDS-NMS and ≥8 points on the HADS for depression were necessary. Similarly, for anxiety treatment consideration, ≥6 points on any item within domain B and ≥8 points on the HADS for anxiety were required. ^c^Number of participants that were treated according to the Swedish guidelines, and percent of those that were symptomatic that were treated according to the Swedish guidelines. ^d^Number of participants that were treated according to the international guidelines, and percentage of those that were symptomatic that were treated according to the international guidelines. ^e^Number of participants that were treated according to the Swedish and/or international guidelines, and percent of those that were symptomatic that were treated according to the Swedish and/or international guidelines. ^f^Presented separate for male and female since there are only treatment guidelines for men. Adherence to treatment guidelines include only male participants. ^g^Rapid eye movement sleep behavior disorder. ^h^The treatment guidelines do not differentiate between physical and mental fatigue. Therefore, adherence to the treatment guidelines consider both types of fatigue in the assessment.

The most prevalent NMS scoring ≥6 points on the MDS-NMS scale were “Muscle, joint or back pain” (65%), “Decreased smell” (59%), and “Urinary urgency” (49%) (Table 2). The NMS that most participants were estimated to require treatment for, and for which treatment guidelines existed, were pain (69%), urinary problems (56%), and cognitive dysfunction (55%) (Table 3). Impulse control disorders (ICD) were the least common symptoms on the MDS-NMS scale, with 0 –2% of participants having ≥6 points on any of the items within the domain. This was followed by “Snoring or difficulties breathing” (3% with ≥6 points) and “Delusions” (4% with ≥6 points) (Table 2). The NMS that had the lowest proportion of participants requiring treatment, and where treatment guidelines existed, were “Nausea or stomach sickness” (9%) and “Dysphagia” (13%) (Table 3).

### Adherence to NMS treatment guidelines

On average, 32% (SD:±23) of NMS were treated in adherence to national or international treatment guidelines (Table 3). Among patients with mild PD (Hoehn and Yahr 1–2), an average of 26% (SD:±21) of NMS were treated in accordance with the guidelines. Patients with moderate PD (Hoehn and Yahr 3) had an average treatment adherence rate of 35% (SD:±26), while those with severe PD (Hoehn and Yahr 4) exhibited an average adherence rate of 39% (SD:±34) to the guidelines. The symptom that exhibited the highest adherence to both international and national guidelines was depression, with 79% of the 29 symptomatic participants receiving treatment in accordance with either Swedish or MDS guidelines. Constipation also demonstrated high adherence to treatment guidelines, with 77% of the 66 symptomatic participants being treated in alignment with Swedish guidelines and 65% adhering to international guidelines.

Dysphagia had the lowest adherence to treatment guidelines, as none of the 22 participants with this symptom received treatment in accordance with the guidelines. Additionally, only 4% of the 28 participants with excessive daytime sleepiness, 7% of the 58 participants experiencing symptomatic apathy, and 7% of the 91 patients experiencing cognitive impairment were treated according to national or international guidelines.

### Treatment for NMS

For cognition, 7% of symptomatic individuals received rivastigmine (clinically useful, priority 4), while 8% were prescribed memantine (priority 9). 56% of the patients experienced symptomatic urinary problems. Out of those, 4% were treated with peripheral anticholinergic medication (priority 6), 20% received mirabegron, 2% were given a low dose of tricyclic antidepressants (TCA), 1% received botulinum toxin injections in the bladder, and 1% were treated with a low dose of antidiuretic hormone. However, 67% of the patients had no treatment for their urinary problems. Among the 114 patients who experienced symptomatic pain, only 43 (38%) received treatment in line with national or international guidelines. The most prescribed medication was safinamide (21%). Oxycodone-naloxone is the only recommended medication in the MDS guidelines, and 5 out of 114 patients (4%) received it. (Tables 4 and 5).

**Table 4:** Pharmacological treatment of non-motor symptoms in Parkinson disease in relation to the Swedish National Board of Health and Welfare’s guidelines and in the Swedish Movement Disorder Society’s treatment guidelines (*n* = 165)^a^

	Total cohort^b^ (*n*, %)	Symptom present^c^ (*n*, %)	Symptomatic individuals^d^ (*n*, %)
Depression
Pramiprexol^e^	84 (51%)	57 of 122 (47%)	14 of 29 (48%)
Antidepressive medication	83 (50%)	63 of 122 (52%)	21 of 29 (72%)
SNRI^Prio3^ *(Venlafaxin)*	42 (25%)	33 of 122 (27%)	13 of 29 (45%)
TCA^Prio4^	3 (2%)	2 of 122 (2%)	1 of 29 (3%)
SSRI^*Pri*^^∘8^	10 (6%)	8 of 122 (7%)	3 of 29 (10%)
NaSSA (Mirtazapin)^f^	39 (24%)	30 of 122 (25%)	10 of 29 (34%)
Anxiety
SNRI	42 (25%)	38 of 127 (30%)	16 of 44 (36%)
SSRI	10 (6%)	7 of 127 (6%)	3 of 44 (7%)
TCA	3 (2%)	2 of 127 (2%)	1 of 44 (2%)
Low dose of benzodiazepine	26 (16%)	24 of 127 (19%)	13 of 44 (30%)
Pregablin	3 (2%)	2 of 127 (2%)	0 of 44 (0%)
Apathy
Piribedil^g^	0 (0%)	0 of 126 (0%)	0 of 58 (0%)
Rivastigmin	7 (4%)	6 of 126 (5%)	4 of 58 (7%)
Psychosis
Atypical antipsychotics	11 (7%)	8 of 83 (10%)	5 of 32 (16%)
Clozapine^Prio3^	4 (2%)	2 of 83 (2%)	2 of 32 (6%)
Quetiapin^Prio7^	7 (4%)	6 of 83 (7%)	3 of 32 (9%)
Cognitive impairment
Cholinesterase inhibitors	7 (4%)	7 of 154 (5%)	6 of 91 (7%)
Rivastigmine^Prio4^	7 (4%)	7 of 154 (5%)	6 of 91 (7%)
Donezepil^Prio4^	0 (0%)	0 of 154 (0%)	0 of 91 (0%)
Galantamine ^Prio4^	0 (0%)	0 of 154 (0%)	0 of 91 (0%)
Memantin^Prio9^	7 (4%)	7 of 154 (5%)	7 of 91 (8%)
Orthostatic hypotension
Etilefrin	5 (3%)	4 of 96 (4%)	3 of 55 (5%)
Midodrine^Prio3^	7 (4%)	5 of 96 (5%)	4 of 55 (7%)
Fludrocortisone^Prio5^	2 (1%)	2 of 96 (2%)	1 of 55 (2%)
Droxidopa ^Prio8^	0 (0%)	0 of 96 (0%)	0 of 55 (0%)
Pyridotigmin	1 (1%)	1 of 96 (1%)	1 of 55 (2%)
Atomoxetin	0 (0%)	0 of 96 (0%)	0 of 55 (0%)
Urinary problems
Peripheral anticholinergic	7 (4%)	6 of 131 (5%)	5 of 93 (5%)
Tolterodin^Prio6^	3 (2%)	2 of 131 (2%)	2 of 93 (2%)
Fesoterodin^Prio6^	1 (1%)	1 of 131 (1%)	1 of 93 (1%)
Solinfenacin^Prio6^	3 (2%)	3 of 131 (2%)	2 of 93 (2%)
Darifenacin ^Prio6^	0 (0%)	0 of 131 (0%)	0 of 93 (0%)
Mirabegron	33 (20%)	33 of 131 (25%)	25 of 93 (27%)
Low dose TCA	3 (2%)	1 of 131 (1%)	1 of 93 (1%)
Botox injection bladder^Prio7^	2 (1%)	2 of 131 (2%)	2 of 93 (2%)
Low dose ADH^h^	2 (1%)	2 of 131 (2%)	1 of 93 (1%)
Drooling of saliva
Local atropine	8 (5%)	8 of 100 (8%)	8 of 48 (17%)
Amantadin/Dinetrel	36 (22%)	24 of 100 (24%)	10 of 48 (21%)
Botox injection parotis^Prio4^	3 (2%)	3 of 100 (3%)	3 of 48 (6%)
Dysphagia
Apomorphine	4 (2%)	0 of 65 (0%)	0 of 22 (0%)
Nausea or stomach sick
COMT inhibitors	59 (36%)	21 of 65 (32%)	6 of 15 (40%)
Entacarpone^*i*^	59 (36%)	21 of 65 (32%)	6 of 15 (40%)
Comtess	0 (0%)	0 of 65 (0%)	0 of 15 (0%)
Tasmar	1 (1%)	1 of 65 (2%)	0 of 15 (0%)
Domperidone	2 (1%)	1 of 65 (2%)	1 of 15 (7%)
Proton pump inhibitors	32 (19%)	14 of 65 (22%)	4 of 15 (27%)
Histamine antagonist	4 (2%)	1 of 65 (2%)	1 of 15 (7%)
Constipation
Laxatives	81 (49%)	71 of 107 (66%)	51 of 66 (77%)
Makrogol	66 (40%)	57 of 107 (53%)	41 of 66 (62%)
Microlax	5 (3%)	4 of 107 (4%)	3 of 66 (5%)
Lactulose	5 (3%)	4 of 107 (4%)	3 of 66 (5%)
Cilaxoral	7 (4%)	7 of 107 (7%)	6 of 66 (9%)
Other	8 (5%)	8 of 107 (7%)	5 of 66 (8%)
Probiotics	5 (3%)	5 of 107 (5%)	3 of 66 (5%)
Insomnia
Mirtazapine	39 (24%)	27 of 107 (25%)	16 of 55 (29%)
Zopiclone	21 (13%)	17 of 107 (16%)	10 of 55 (18%)
Melatonin	9 (5%)	7 of 107 (7%)	4 of 55 (7%)
Extended release levodopa/DA^j^	67 (41%)	43 of 107 (40%)	25 of 55 (45%)
Mianserin^k^	15 (9%)	8 of 107 (7%)	3 of 55 (5%)
REM sleep disturbances
Clonazepam	19 (12%)	15 of 95 (16%)	7 of 30 (23%)
Melatonin	9 (5%)	5 of 95 (5%)	1 of 30 (3%)
Restless leg syndrome
Antiepileptics	14 (8%)	4 of 82 (5%)	2 of 53 (4%)
Gabapentin	11 (7%)	4 of 82 (5%)	2 of 53 (4%)
Pregabalin	3 (2%)	0 of 82 (0%)	0 of 53 (0%)
Oxycodone-Naloxone	3 (2%)	1 of 82 (1%)	1 of 53 (2%)
Zoplikone	21 (13%)	9 of 82 (11%)	5 of 53 (9%)
Clonazepam	19 (12%)	14 of 82 (17%)	11 of 53 (21%)
Pain
Apomorphine	4 (2%)	4 of 133 (3%)	4 of 114 (4%)
Rotigotine	5 (3%)	5 of 133 (4%)	4 of 114 (4%)
Safinamide	36 (22%)	28 of 133 (21%)	24 of 114 (21%)
Opioids	15 (9%)	14 of 133 (11%)	13 of 114 (11%)
Oxycodone-Naloxone	5 (3%)	5 of 133 (4%)	5 of 114 (4%)
Other	11 (7%)	10 of 133 (8%)	9 of 114 (8%)
Gabapentin	10 (6%)	9 of 133 (7%)	9 of 114 (8%)
Amitriptyline	3 (2%)	3 of 133(2%)	3 of 114 (3%)
Pregablin	3 (2%)	3 of 133 (2%)	3 of 114 (3%)
Dystonia
Botox	8 (5%)	7 of 92 (8%)	7 of 49 (14%)
Excessive sweating
Propranolol	9 (5%)	2 of 67 (3%)	1 of 39 (3%)
Mirtazapine	39 (24%)	18 of 67 (27%)	9 of 39 (23%)
Anticholinergics	8 (5%)	3 of 67 (4%)	3 of 39 (8%)

SNRI, serotonin–norepinephrine reuptake inhibitors; TCA, tricyclic antidepressants; SSRI, selective serotonin reuptake inhibitor; NaSSA, noradrenergic and specific serotonergic antidepressants; TeCA, tetracyclic antidepressants; DA, dopamine agonist; REM, rapid eye movement sleep behavior disorder. ^a^Description of how the patients is treated in relation to the Swedish guidelines. Priority 0–4 (recommended/should be used). Priority 5–7 (can be used). Priority 8–10 (can be used as an exception) ^b^Number of participants of the total cohort (*n* = 165) that have the treatment, and percent of the total cohort that have the treatment. ^c^Number of participants with the symptoms that have the treatment. Defined as ≥1 point on that MDS-NMS item if there is a specific treatment for the item. If there are no designated treatments for the specific item but for the entire domain, it is defined as ≥1 points on any item within that domain. The patient has the symptom insomnia, if they have ≥1 point on the insomnia item (domain K question 1) in the MDS-NMS and ≥1 point on the PDSS-2. They have the symptom daytime sleepiness if they have ≥1 point on the daytime sleepiness item (domain K question 3) in the MDS-NMS and ≥1 points on the ESS. They have the symptom depression if they have ≥1 points on any item within domain A in MDS-NMS and ≥1 points on the HADS for depression. Similarly, for the symptom anxiety, ≥1 point on any item within domain B and ≥1 on the HADS for anxiety is required. ^d^Number of participants who are considered to require treatment for the symptoms that have the treatment. Patients with ≥6 points on that item on the International Parkinson and Movement Disorder Society - Non-Motor Rating Scale (MDS-NMS) is considered to require treatment for that symptom. If there is no designated treatment for a specific item but for the entire domain, the patient needed to score ≥6 points on any item within that domain. To be considered treatment for insomnia, a patient needs ≥6 points on the insomnia item (domain K question 1) in the MDS-NMS and ≥15 points on the PDSS-2. Daytime sleepiness requires ≥6 points on the daytime sleepiness item (domain K question 3) in the MDS-NMS and ≥13 points on the ESS. In the depression category, ≥6 points on any item within domain A in MDS-NMS and ≥8 points on the HADS for depression are necessary. Similarly, for anxiety treatment consideration, ≥6 points on any item within domain B and ≥8 points on the HADS for anxiety are required. ^e^It is unknown whether the indication for initiating pramipexole was depression. ^f^Recommended if the patient has sleep disturbances as well. ^g^Recommended after STN-DBS. ^h^Recommended if the patient has nocturia as well. ^i^It is unknown whether the indication for initiating entacapone was nausea. ^j^Recommended if the patient has sleep disturbances due to PD symptoms during nighttime. All patients were asked about the reason for their sleep disturbances. ^k^Recommended if the patient is included for depression as well.

**Table 5:** Pharmacological treatment of non-motor symptoms in Parkinson disease in relation to the Parkinson and Movement Disorder Society guidelines (*n* = 165)^a^

	Total cohort^b^ (*n*, %)	Symptom present^c^ (*n*, %)	Symptomatic individuals^d^ (*n*, %)
Depression
Pramiprexol^Clinuseful,e^	84 (51%)	57 of 122 (47%)	14 of 29 (48%)
Antidepressive medication	54 (33%)	42 of 122 (34%)	16 of 29 (55%)
SNRI (Venlafaxin) ^Clinuseful^	42 (25%)	33 of 122 (27%)	13 of 29 (45%)
TCA^Pos useful^	3 (2%)	2 of 122 (2%)	1 of 29 (3%)
SSRI^*Pos* *useful*^	10 (6%)	8 of 122 (7%)	3 of 29 (10%)
Apathy
Piribedil^f^	0 (0%)	0 of 126 (0%)	0 of 58 (0%)
Rivastigmin	7 (4%)	6 of 126 (5%)	4 of 58 (7%)
Psychosis
Atypical antipsychotics	11 (7%)	8 of 83 (10%)	5 of 32 (16%)
Clozapine^Clinuseful^	4 (2%)	2 of 83 (2%)	2 of 32 (6%)
Quetiapine^Pos useful^	7 (4%)	6 of 83 (7%)	3 of 32 (9%)
Pimvanserin^Clinuseful^	0 (0%)	0 of 83 (0%)	0 of 32 (0%)
Cognitive impairment
Cholinesterase inhibitors	7 (4%)	7 of 154 (4%)	6 of 91 (7%)
Rivastigmine^Clinuseful^	7 (4%)	7 of 154 (4%)	6 of 91 (7%)
Donezepil^Pos useful^	0 (0%)	0 of 154 (0%)	0 of 91 (0%)
Galantamine^Pos useful^	0 (0%)	0 of 154 (0%)	0 of 91 (0%)
Orthostatic hypotension
Midodrine^Pos useful^	7 (4%)	5 of 96 (5%)	4 of 55 (7%)
Fludrocortisone^Pos useful^	2 (1%)	2 of 96 (2%)	1 of 55 (2%)
Droxidopa^Pos useful^	0 (0%)	0 of 96 (0%)	0 of 55 (0%)
Urinary problems
Solinfenacin^Pos useful^	3 (2%)	3 of 131 (2%)	2 of 93 (2%)
Sexual dysfunction^f^
Sildenafil^Clinuseful^	9 (11%)	6 of 29 (21%)	6 of 16 (38%)
Drooling of saliva
Botox injection in parotis^Clinuseful^	3 (2%)	3 of 100 (3%)	3 of 48 (6%)
Glycopyrrolate^Pos useful^	0 (0%)	0 of 100 (0%)	0 of 48 (0%)
Nausea or stomach sick
Domperidone^Pos useful^	2 (1%)	1 of 65 (2%)	1 of 15 (7%)
Constipation
Laxiatives	66 (40%)	57 of 107 (53%)	41 of 66 (62%)
Macrogol^Pos useful^	66 (40%)	57 of 107 (53%)	41 of 66 (62%)
Lubiprostone ^Pos useful^	0 (0%)	0 of 107 (0%)	0 of 66 (0%)
Probiotic and probiotic fiber ^Clinuseful^	5 (3%)	5 of 107 (5%)	3 of 66 (5%)
Insomnia
Rotigotine ^Pos useful^	7 (4%)	3 of 107 (3%)	2 of 55 (4%)
Zoplikone ^Pos useful^	21 (13%)	17 of 107 (16%)	10 of 55 (18%)
Melatonin ^Pos useful^	9 (5%)	7 of 107 (7%)	4 of 55 (7%)
Excessive daytime sleepiness
Modafinil^Pos useful^	5 (3%)	4 of 101 (4%)	1 of 28 (4%)
Pain
Oxycodone-Naloxone^Pos useful^	5 (3%)	5 of 133 (4%)	5 of 114 (4%)
Fatigue^g^
Rasagaline^Pos useful^	35 (21%)	25 of 117 (21%)	15 of 76 (20%)

SNRI, serotonin–norepinephrine reuptake inhibitors; TCA, tricyclic antidepressants; SSRI, selective serotonin reuptake inhibitor ^a^Description of how the patients is treated in relation to the Movement Disorder Society guidelines. Priority 0–4 (recommended/should be used). Pos useful = Possibly useful. Clin useful = Clinically useful. ^b^Number of participants of the total cohort (*n* = 165) that have the treatment, and percent of the total cohort that have the treatment. ^c^Number of participants with the symptoms that have the treatment. Defined as ≥1 point on that MDS-NMS item if there is a specific treatment for the item. If there are no designated treatments for the specific item but for the entire domain, it is defined as ≥1 points on any item within that domain. The patient has the symptom insomnia, if they have ≥1 point on the insomnia item (domain K question 1) in the MDS-NMS and ≥1 point on the PDSS-2. They have the symptom daytime sleepiness if they have ≥1 point on the daytime sleepiness item (domain K question 3) in the MDS-NMS and ≥1 points on the ESS. They have the symptom depression if they have ≥1 points on any item within domain A in MDS-NMS and ≥1 points on the HADS for depression. Similarly, for the symptom anxiety, ≥1 point on any item within domain B and ≥1 on the HADS for anxiety is required. ^d^Number of participants who are considered to require treatment for the symptoms that have the treatment. Patients with ≥6 points on that item on the International Parkinson and Movement Disorder Society - Non-Motor Rating Scale (MDS-NMS) is considered to require treatment for that symptom. If there is no designated treatment for a specific item but for the entire domain, the patient needed to score ≥6 points on any item within that domain. To be considered treatment for insomnia, a patient needs ≥6 points on the insomnia item (domain K question 1) in the MDS-NMS and ≥15 points on the PDSS-2. Daytime sleepiness requires ≥6 points on the daytime sleepiness item (domain K question 3) in the MDS-NMS and ≥13 points on the ESS. In the depression category, ≥6 points on any item within domain A in MDS-NMS and ≥8 points on the HADS for depression are necessary. Similarly, for anxiety treatment consideration, ≥6 points on any item within domain B and ≥8 points on the HADS for anxiety are required. ^e^It is unknown whether the indication for initiating pramipexole was depression. ^f^Since there are only guidelines for men, only men are included here (*n* = 80) ^g^Includes both mental and physical tiredness since the symptoms are not separated in the guidelines.

Out of the 29 individuals with symptomatic depression, 48% received pramipexole (clinically useful), and 72% were prescribed antidepressant medication. Among those receiving antidepressant therapy, 62% were administered venlafaxine (priority 3), 5% were given TCA (priority 4), 14% were prescribed selective serotonin reuptake inhibitors (SSRI) (priority 8), and 48% received mirtazapine. Out of the 66 symptomatic participants with constipation, 51 received laxatives. Macrogol was the most used laxative, accounting for 80% of the cases. Six of the 16 men that experienced symptomatic sexual dysfunction received sildenafil (clinically useful) (Tables 4 and 5).

## DISCUSSION

The primary finding of this study was that among PD patients with a Hoehn and Yahr stage of ≤4, treatment of multiple NMS was limited. On average, only 32% of NMS were treated in adherence to national or international treatment guidelines. Depression showed the highest adherence to guidelines (79%), followed by constipation (77%), while dysphagia had the lowest adherence (0%). These findings highlight the need for improved screening and treatment of NMS among PD patients.

Pain was the most prevalent NMS, with 69% of the participants experiencing symptomatic pain (≥6 points on item 1, 2, or 3 within domain L) and 81% reporting pain symptoms (≥1 point on item 1, 2, or 3 within domain L). This aligns with previous studies showing that 68–95% of PD patients encounter pain-related issues [26]. Pain in PD is often multi-factorial and even though PD is not always the main source of pain, it is often amplified by motor and non-motor PD symptoms [26]. Only 38% of patients received appropriate pain treatment in accordance with the guidelines. However, 27% of patients received pain treatment outside the guidelines, such as paracetamol, or non-steroidal anti-inflammatory drugs. It is possible that these drugs were suitable for specific patients whose primary pain issue was unrelated to PD. Moreover, patients’ ratings indicated that nonsteroidal anti-inflammatory drugs were reported as the most effective analgesic medication [27], despite not being included in the guidelines. This suggests that the guidelines may not address the diverse pain experiences in PD. Optimizing dopaminergic treatment is important for managing PD-related pain [26],but this aspect was not examined in this study due to limitations in assessing treatment optimization over the phone. As specific pain types couldn’t be identified during the phone interviews, any guideline-based treatment was considered appropriate, despite varying guidelines for different pain types. Consequently, less than 38% of patients may have received optimal treatment. These findings indicate a need for physicians to improve recognition and management of pain in PD patients. Further research is warranted to identify effective pain treatments for PD and to enhance the current guidelines.

Consistent with earlier research [28], urinary problems were found to be a prevalent NMS, and 56% of the participants were defined to require treatment. However, only 31 out of 93 patients received treatment in accordance with national or international guidelines. The development of urinary problems has been linked to a notable decline in quality of life [14]. Hence, improved screening and effective management of urinary problems are crucial to enhance patients’ overall well-being. Only 0–2% of patients scored ≥6 points on any item within the “Impulse control disorder” domain. However, previous studies indicate an occurrence rate of approximately 14% for ICD among patients [29]. The limited reporting of ICD symptoms might stem from feelings of embarrassment or reluctance to discuss such issues. Given the significant impact of ICD on quality of life and functioning [29], uncovering these problems is crucial. Encouraging open discussions during clinician meetings, especially with established relationships, could encourage greater openness. Additionally, involving a patient’s relative in the conversation might provide further insights.

Depression and constipation had the highest adherence rates to treatment guidelines, with 79% and 77% compliance, respectively. Among the 29 participants with symptomatic depression, the most common treatments were venlafaxine (45%), mirtazapine (34%), and pramipexole (48%). Pramipexole is prescribed for both depression and motor symptoms, making it unclear if it was specifically intended for depression. Additionally, 10% of symptomatic patients received SSRI, despite only being recommended as an exception according to national guidelines due to contradictory results [10]. However, international guidelines consider SSRI as “possibly useful” for depression treatment [11]. Even though venlafaxine and pramipexole are more strongly recommended in international guidelines it can therefore be argued that the use of SSRI has some support.

Our findings revealed that only 7% of patients reporting cognitive impairment based on the MDS-NMS scale received guideline-based treatment. Often, memantine was prescribed instead of cholinesterase inhibitors, despite being a lower priority in Swedish guidelines and not recommended by the MDS guidelines [7, 9–11]. No validated screening scale for cognitive impairment was utilized in this study, which introduces uncertainty regarding the prevalence of cognitive impairment among participants. It is possible that patients both overestimate and underestimate their problems on the MDS-NMS scale. Cognitive impairment is up to six times more common in individuals with PD than in the healthy population [30]. Over 80% of PD patients progress to dementia in later stages, and around 40% of early stage PD patients experience mild cognitive impairment [31]. Our results indicate that 55% had symptomatic cognitive impairment, aligning well with those numbers, despite relying solely on the MDS-NMS scale. Early identification and targeted treatment of mild cognitive dysfunction is crucial in order to improve cognitive reserve and protect cognitive status [31]. Consequently, it is likely that many of the patients that experienced cognitive problems according to the MDS-NMS scale should be considered for treatment or at least undergo an assessment for cognitive impairment. However, it is important to note that the decision for treatment should not be based solely on the MDS-NMS scale.

Despite available clinical useful treatment, only 38% of men with sexual dysfunction received an appropriate treatment. This finding aligns with previous research indicating that sexual dysfunction is often neglected among PD patients [32]. This undertreatment might be attributed to physicians struggling to address the issue and patients feeling uncomfortable discussing it. Only 20% of men reported sexual dysfunction, although research suggests it affects up to 82% of men with PD, and is about twice as common as in aged matched controls without PD [33]. This indicates a significant stigma surrounding the topic, leading patients to avoid discussing it even when prompted with a direct question. An active and satisfying sex life is associated with improved quality of life and better motor and NMS control in men with PD [34, 35]. Thus, clinicians should probably improve their ability to discuss and address sexual dysfunction in a sensitive and supportive manner, to ensure that patients feel comfortable and receive appropriate treatment and support. Currently, there are no recommended treatments for sexual dysfunction in national guidelines, and international guidelines lack specific recommendations for addressing sexual dysfunction in women with PD, despite its negative impact on their quality of life [36]. However, treatments such as menopause hormone therapy, local estrogen therapy, and vaginal dehydroepiandrosterone are available for women with sexual dysfunctions [37]. Studies on their effectiveness in women with PD are necessary to update the guidelines and provide recommendations for both sexes. Additionally, national guidelines should be revised to include treatment options for sexual dysfunction, aiming to improve the quality of life for PD patients experiencing this symptom.

Some of the symptoms with the lowest adherence to guidelines were apathy (4 out of 58 patients), excessive daytime sleepiness (1 out of 28 patients), and dysphagia (0 out of 22 patients). However, the recommended treatments for these symptoms are based on limited evidence or only a few studies[7, 11]. For apathy, rivastigmine is recommended, and piribedil is recommended after subthalamic nucleus deep brain stimulation (DBS). However, according to the MDS guidelines, both rivastigmine and piribedil have only been evaluated in one positive, small-sized, but high-quality study [11]. The national guidelines also indicate limited evidence for the treatment of apathy[7]. Regarding dysphagia, there are no available international guidelines, and according to the Swedish guidelines, apomorphine may offer temporary relief [7]. Only the MDS guidelines provide recommendations for excessive daytime sleepiness, and while modafinil is considered “possibly useful,” there is insufficient evidence to determine its effectiveness conclusively [11].

Previous studies have revealed that neurologists fail to identify NMS in over 50% of consultations [38], despite the fact that NMS have been shown to have a greater impact on the quality of life of PD patients compared to motor symptoms [39]. In the assessment of NMS, scales such as the NMS Scale (NMSS) and NMSQ are often used [17, 40]. LeWitt et al. [40] examined unmet needs for both motor symptoms and NMS in PD. They identified a lack of unified guidelines for incorporating both patient-completed questionnaires like the NMSQ and clinician-completed tools like the NMSS or MDS-NMS for routine assessment in clinical settings, resulting in NMS often being overlooked and underdiagnosed. Additionally, they identified an unmet need for individualized NMS burden grading to guide personalized management. Moreover, all prevalent and dominant NMS could be shown to be poorly treated, and for many NMS, such as anxiety, apathy and urinary dysfunction, there was no strong evidence for how NMS should be managed the best possible way. These findings align with the results of our study, indicating a frequent oversight and inadequate treatment of NMS, with a limited adherence to treatment guidelines.

The perception of distress or disturbance caused by a symptom can vary significantly among individuals, making it difficult to establish definitive criteria for determining the need for treatment. However, it can be argued that if a patient finds a symptom distressing, it is important to address and alleviate their concerns. In the case of the MDS-NMS scale, there is no predetermined cut-off value. We selected a cutoff of ≥6 to determine when treatment is needed based on the rationale that a score of at least 6 indicates either minor distress or disturbance with frequent problems, or problems occurring sometimes but causing considerable distress or disturbance. In both cases, it can be argued that treatment could be beneficial. Sometimes, treatment for a specific symptom is recommended only if the patient presents with certain accompanying problems. For instance, mirtazapine is recommended for depression treatment when the patient also experiences sleep disturbances. Additionally, the use of low-dose antidiuretic hormone is specifically recommended for patients with nocturia. We considered these recommendations when evaluating adherence to treatment guidelines.

There are several reasons why clinicians may not adhere to NMS treatment guidelines, including prior unsuccessful attempts with the prescribed drug due to ineffectiveness or severe side effects. Additionally, the clinicians’ past experiences might lead them to believe that certain recommended treatments are ineffective, prompting them to avoid them. Also, some patients may experience multiple side effects or be reluctant to take additional medication, while others may be at risk for drug interactions with other medications. Furthermore, some clinicians might prefer to steer clear of polypharmacy. To reduce the risk of polypharmacy while effectively managing patients’ NMS, one approach could be to conduct a thorough assessment of the NMS experienced by the patients and the degree to which they are affected by the NMS, utilizing tools such as the MDS-NMS. This approach enables clinicians to prioritize treatment based on symptom prevalence and subjective severity. Continuous evaluations of both treatment and NMS are also essential, allowing for withdrawal attempts if deemed appropriate.

On average, 32% of NMS were treated in adherence with guidelines for the entire group. There was a trend towards a higher percentage of NMS treated in adherence with guidelines in patients with more severe PD (26% for mild PD, 35% for moderate PD, and 39% for severe PD). One possible reason for the increased adherence to guidelines when treating NMS in more severe disease staged could be a higher awareness of the treating clinician to NMS in those patients as follow-up times might be longer, which provides more patient contact. Furthermore, patients with more severe disease generally exhibited more pronounced NMS, as indicated by a total MDS-NMS score of a median 88 (IQR: 60–118) for mild PD, 119 (IQR: 81–170) for moderate PD, and 133 (IQR: 96–161) for severe PD (data not shown). More pronounced NMS, both reported and visible, is likely to prompt the clinician to initiate treatment. Another explanation could be that clinicians prioritize optimizing dopaminergic treatment during the early stages, rather than introducing additional medications. Optimizing dopaminergic treatment is often recommended before introducing other medications. In some cases, clinicians might be focusing on optimizing dopaminergic treatment or evaluating NMS again after optimizing dopaminergic treatment, which could explain the absence of specific NMS treatments. Furthermore, alternative interventions such as DBS or physiotherapy may have been pursued. For instance, while none of the 22 patients with dysphagia received appropriate pharmacological treatment for this symptom, it is likely that some of them received alternative interventions such as help from a logopedic or gastrostomy to address their swallowing difficulties. Moreover, it is possible that some of the patients with dystonia underwent a DBS procedure, which is also recommended. This study focused on the pharmacological aspects of NMS treatment. However, future research should explore the non-pharmacological aspects of NMS treatment to provide a comprehensive understanding of effective interventions.

A limitation of this study is that some patients may have been symptomatic before receiving treatment but are no longer symptomatic, which could impact the results. However, Table 4 and 5 provide valuable information regarding the number of patients receiving specific treatments, the proportion of patients with a symptom receiving treatment, and the proportion of symptomatic patients receiving each treatment. If patients undergoing treatment have a symptom but are not symptomatic, it sometimes suggests improvement due to treatment. Additionally, incomplete documentation of medical treatments by clinicians may have resulted in the omission of certain treatments from the analysis. The specific indications for prescribing certain treatments are also unclear, such as pramipexole, which can be effective for both depression and motor problems, and catechol-O-methyltransferase (COMT) inhibitors, which has an impact on both motor symptoms and abdominal discomfort. Moreover, the NMS-MDS questionnaire was administered via telephone and translated into Swedish, introducing the potential for translation inconsistencies and varying interpretations of the questions. To address this, a single rater (CJ) conducted all the interviews to maintain translation consistency. Moreover, we included patients with a Hoehn and Yahr stage ≤4, however, 72% of included patients were either in Hoehn and Yahr stage 2 or 3. Importantly, we therefore further analyzed the adherence to guidelines depending on the HY stage. The average number of NMS that were treated in accordance with guidelines was slightly higher for patients with more severe PD, indicating there might be some variations between different disease stages. However, Rosqvist et al. [12] focused on adherence in PD patients with late-stage PD (Hoehn and Yahr ≥4), complementing our study and providing a comprehensive overview of the entire patient group. It is also worth noting that some of the symptoms were only reported by a small number of participants, limiting the ability to draw definitive conclusions from the results. However, these findings still provide valuable insights into the potential inadequacies in the treatment of specific symptoms and highlight the need for further investigation into adherence to guidelines.

In conclusion, this study confirms the high prevalence of NMS among PD patients across the motor severity spectrum. In median, each patient experienced 14 NMS and required treatment for seven different NMS. Moreover, there was a low adherence to national and international pharmacological treatment guidelines for NMS. To optimize the treatment of NMS and improve the quality of life for individuals with PD, it is crucial to enhance the detection of NMS. This can be accomplished by incorporating tools like NMSQ or similar questionnaires regularly during clinical assessments. Furthermore, it is essential to advance our understanding of effective NMS treatment strategies. This can be achieved by enhancing adherence to existing NMS treatment guidelines and by further evaluating and refine these guidelines to enhance their effectiveness. Further research is necessary to explore more effective methods of treating NMS with fewer side effects and drug interactions, as well as developing reliable ways to detect NMS.

## Data Availability

The data supporting the findings of this study are available on request from the corresponding author. The data is not publicly available due to privacy or ethical restrictions.
